# Service redesign interventions to reduce waiting time for paediatric rehabilitation and therapy services: A systematic review of the literature

**DOI:** 10.1111/hsc.13866

**Published:** 2022-06-18

**Authors:** Katherine E. Harding, Chantal Camden, Annie K. Lewis, Kadija Perreault, Nicholas F. Taylor

**Affiliations:** ^1^ La Trobe University, School of Allied Health Human Services and Sport Melbourne Victoria Australia; ^2^ Eastern Health Allied Health Clinical Research Office Box Hill Victoria Australia; ^3^ Sherbrooke University, School of Rehabilitation Sherbrooke Québec Canada; ^4^ Center for Interdisciplinary Research in Rehabilitation and Social Integration, Centre intégré universitaire de santé et de services sociaux de la Capitale‐Nationale Québec City Québec Canada; ^5^ Department of Rehabilitation, Faculty of Medicine Université Laval Québec City Québec Canada

**Keywords:** access to care, community care, Paediatrics, rehabilitation, service delivery and organisation

## Abstract

Despite well‐documented benefits of rehabilitation and therapy services for children with disabilities, long waiting lists to access these services are common. There is a growing body of evidence, primarily from mixed or adult services, demonstrating that waiting times can be reduced through strategies that target wasteful processes and support services to keep up with demand. However, providers of rehabilitation and therapy services for children face additional complexities related to the long‐term nature of many developmental conditions and the need to consider timing of interventions with developmental milestones and education transition points. This review aimed to synthesise available evidence on service redesign strategies in reducing waiting time for paediatric therapy services. We conducted a systematic review of studies conducted in outpatient paediatric rehabilitation or therapy settings, including physical and mental health services, evaluating a service redesign intervention and presenting comparative data on time to access care. Two reviewers independently applied inclusion criteria, assessed risk of bias and extracted data. Findings were analysed descriptively and the certainty of evidence was synthesised according to criteria for health service research. From 1934 studies identified, 33 met the criteria for inclusion. Interventions were categorised as rapid response strategies, process efficiency interventions or substitution strategies (using alternative providers in place of medical specialists). Reductions in waiting time were reported in 30 studies. Evidence is limited by study designs with high risk of bias, but this is mitigated by consistency of findings and large effect sizes. There is moderate‐certainty evidence that service redesign strategies similar to those used in adult populations can be applied in paediatric rehabilitation and therapy settings to reduce waiting time.

**What is known about the topic?**

- Delays to access paediatric therapy and rehabilitation services are a major problem.
- Service redesign interventions can improve access, but previous reviews have focussed on services for adults or mixed populations.
- Evidence generated in other settings may not be directly applicable to children's services.

**What this paper adds?**

- Service redesign initiatives—including process improvement approaches, rapid response interventions and strategies that use trained allied health in place of medical specialists for some assessments—can reduce waiting lists in paediatric settings.
- These findings are similar to those observed in adult/mixed settings.
- Evidence is limited by low‐quality studies; future studies using more rigorous designs are required to guide service providers on the best use of limited resources to reduce waiting times.

## 1 INTRODUCTION

Children with disabilities and chronic health conditions require timely access to services to minimise impairments, activity limitations and/or participation restrictions, and maximise their quality of life. The right to rehabilitation and therapy interventions is recognised in the United Nations Convention on the Rights of Persons with Disabilities, and should ideally be timed to take advantage of the optimal periods for intervention during a child's growth and development (Novak, 2014).

Long waiting lists for speech and language therapy, occupational therapy, physiotherapy, psychology and other services that offer rehabilitation or therapy for children with disabilities and chronic health conditions are common (Feldman, 2008; A. R. Millar, 2008). This is despite evidence that neuroplasticity of the developing brain provides optimal periods in which targeted intervention is most effective to maximise function and reduce secondary impairments (Novak, 2014). Delayed access to rehabilitation and therapy services for children with disabilities who miss this window of opportunity can therefore lead to poorer outcomes and contribute to increased stress on families (Feldman, 2008). Access delays can also contribute to demoralisation among service providers and act as a deterrent to referral (Keating, 1998). Short waiting times are also among the factors most highly valued by families in the delivery of these services (Gallego et al., 2018), and access issues disproportionately affect children experiencing social and economic disadvantage (Porterfield & McBride, 2007).

Waiting times for health services can be reduced by implementing strategies that target wasteful processes and alter models of care delivery to help services to keep up with demand (Kreindler, 2008). These types of interventions could be described as service redesign, which has been broadly defined as a process change that aims to achieve ‘speedy and effective care’ by identifying and removing sources of delays or potential for error (Locock et al., 2001). However, much of the evidence for service redesign interventions for reducing waiting time comes from services for adults or mixed populations (Harding et al., 2018; Murray & Berwick, 2003) and applicability to paediatric services cannot be assumed. Services for children, and particularly those with developmental or long‐term conditions, may need to take into account unique features that do not apply in adult settings. For example, service delivery systems may need to be structured around key points of transition within the education system. In addition, the need to intervene at specific developmental stages and the roles of families may add other complicating and interacting factors to designing access systems.

This systematic review of the literature aimed to synthesise the literature on service redesign initiatives to reduce waiting time for paediatric services. Specifically, we sought to find out (1) whether service redesign strategies are effective in reducing waiting time for paediatric therapy services and (2) whether some types of strategies are more effective than others in achieving this goal.

## 2 METHOD

This systematic review was registered prospectively with PROSPERO (CRD42019140963) and reported according to the Preferred Reporting Items for Systematic Reviews and Meta‐Analyses (PRISMA) guidelines (http://prisma‐statement.org/prismastatement/Checklist.aspx).

### 2.1 Search strategy

A search strategy was designed to identify studies that evaluated service redesign strategies aiming to reduce waiting time for paediatric rehabilitation or therapy services. Medline, Embase, Psych Info and CINAHL were searched from the earliest available date until March 2021.

The search strategy used the three concepts of ‘paediatric’, ‘therapy services’, and ‘waiting’. Synonyms and related terms for each concept were combined using ‘OR’ and all three searches were then combined using ‘AND’. To capture literature within the broad definition of rehabilitation or therapy services, we included a range of search terms including individual allied health professions (such as physical therapy and occupational therapy), conceptual terms associated with these services such as disability, mental health and rehabilitation, and common health conditions seen in these services such as cerebral palsy, autism and developmental delay. Due to the broad nature of the concept of ‘waiting’, searches were done using the MeSH terms ‘waiting list’ and ‘appointments and schedules’ and terms related to ‘wait’ and ‘time’ were combined using proximity operators. For example, “Wait adj5 time” was used to identify “time spent waiting” or “waiting time” and “wait* adj5 number” to find “number of patients waiting” or “number on the waitlist”. An example of the search strategy can be found in Appendix S1.

Reference lists of selected studies were scanned for relevant articles, and citation tracking of included papers completed in Google Scholar.

### 2.2 Inclusion and exclusion criteria

Studies were included from settings that provided rehabilitation or therapy services to children within the community, investigated the effectiveness of a service redesign intervention and reported data on an outcome related to timeliness of care, such as time to first appointment or the size of the waiting list.

For the purpose of the review, service redesign was defined as any intervention that changes the process by which a service is delivered to achieve timely and effective care. This could include acceptance of referrals, appointments and scheduling, the personnel delivering the intervention, the style of intervention (group/individual, frequency/intensity of therapy) or location of intervention (home vs. clinic/school, telehealth). Paediatric rehabilitation or therapy was broadly defined as a service provided by members of the allied health professions, such as physiotherapy, occupational therapy, speech and language therapy or psychology, either alone or within the context of a multi‐disciplinary team, to reduce impairments, activity limitations, and/or participation restrictions for children with disabilities, mental health conditions, developmental delays or chronic health conditions.

Any research design was acceptable, provided quantitative data were reported that compared either waiting time from referral to first appointment or the size of a waiting list under different service designs. For example, eligible designs could include randomised trials allocating patients to different models of care, studies presenting data before and after an intervention or comparisons of different sites with different models of care. Only peer‐reviewed papers were included, published in either English or French. Inclusion and exclusion criteria are presented in more detail in Appendix S2.

### 2.3 Article selection and data extraction

Two of a pool of five reviewers independently reviewed the title and abstracts of each retrieved study against selection criteria using the online platform Covidence (http://app.covidence.org). Full‐text articles were retrieved for all studies that could not be excluded based on screening of titles and abstracts, and these were assessed against inclusion criteria. Discrepancies were resolved either by consensus or with the assistance of a third reviewer if required. Data were extracted using a form designed within Covidence, which included study characteristics (setting, design and population), nature of intervention and comparison condition, outcomes reported, waiting time outcomes and any secondary outcomes reported (such as patient outcomes, satisfaction and other service outcomes such as failure‐to‐attend rates and costs).

### 2.4 Risk of bias

Risk of bias for each included study was assessed independently by two of a pool of five researchers using six of the 11 internal validity items of the Downs and Black checklist for the assessment of methodological quality of randomised and non‐randomised studies in healthcare (Downs & Black, 1998). The items chosen were those that were most relevant to the study designs in the selected studies: (1) whether participants were randomised to intervention and comparison groups; (2) whether groups being compared were recruited from the same population; (3) whether groups being compared were recruited over the same time period; (4) whether appropriate statistical tests were used to evaluate findings; (5) whether outcomes measures were valid and reliable (awarded ‘yes’ if the measurement of waiting was clearly defined); and (6) whether confounding variables had been considered in the analysis. Other internal validity items on this checklist were considered less useful in differentiating the quality of studies in this review due to the nature of the interventions being studied. For example, waiting list interventions are usually applied to a whole service and those receiving the service are often unaware that a trial is in progress, thus limiting the applicability of concepts such as blinding of participants and compliance with the intervention. Decision rules for the application of each of the six included items are presented in Appendix S3. Results were compared and disagreements resolved through discussion. Studies were not excluded based on the results of risk of bias assessment, but risk of bias was considered in the interpretation of findings.

### 2.5 Analysis

Data were extracted from each included study independently by two of a pool of five reviewers and organised into tables and graphs for comparison and synthesis. Extracted data included the study characteristics, research design, details of the intervention, outcomes reported and key findings. Common themes in the interventions were identified; these themes were then used to categorise the studies into groups based on intervention type. The protocol included provision for meta‐analyses if there were sufficient homogeneity in design and context of the included papers, and minimum data requirements (such as inclusion of means, standard deviations and sample sizes) were consistently reported.

An overall evaluation of the certainty of evidence for the effectiveness of different groups of interventions for reducing waiting time was made following guidelines published by the Evidence‐based Practice Centre (EPC) supported by the Agency for Healthcare Research and Quality (Berkman et al., 2015). The body of literature was judged on the domains of study limitations, directness of the evidence, consistency of findings, precision of results and reporting bias. Where studies were combined to assess certainty of a body of literature, the starting point was considered to be low for observational studies, with the opportunity to upgrade the strength of the evidence based on evaluations of the five domains.

## 3 RESULTS

**FIGURE 1:**
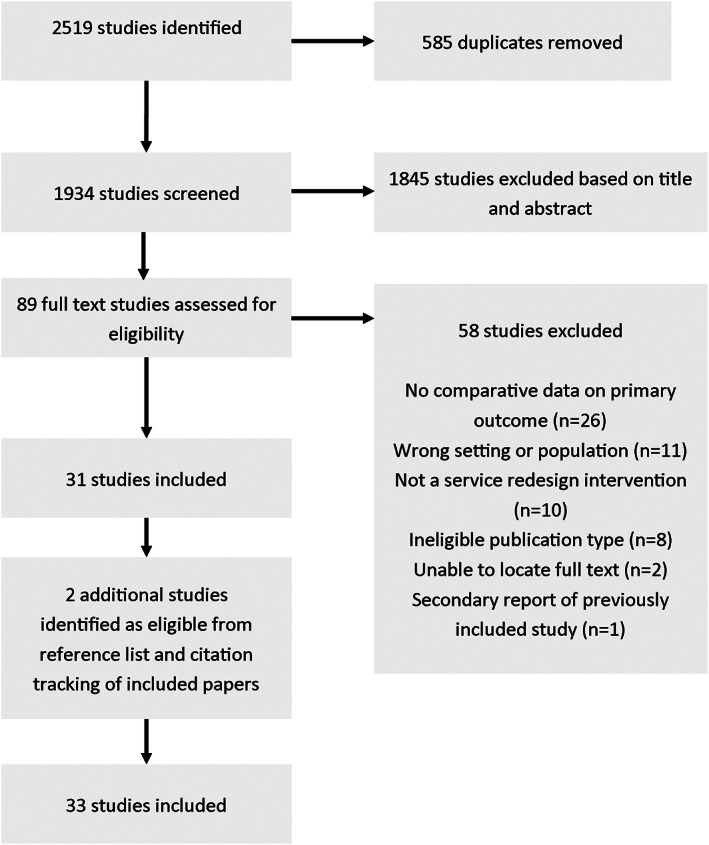
Selection of studies.

The search yielded 1934 studies after removal of duplicates, including an additional two articles identified through citation and abstract checking (Figure 1). A total of 1843 articles were excluded following title and abstract review. For the remaining 91 papers, the full texts were assessed against inclusion criteria. Of these, a further 57 were excluded because they lacked comparative data on waiting time (*n* = 26), no service redesign was described (e.g. studies that examined factors associated with wait times or the impact of wait times on patient outcomes, *n* = 10), the setting was not outpatient therapy and/or paediatrics (such as studies that considered elective surgery or transplant waiting lists, *n* = 11), the publication type was a conference abstract or otherwise ineligible for inclusion (*n* = 8) or the full text was unavailable (*n* = 2). A total of 33 studies were selected for inclusion.

All of the selected studies were from English‐speaking countries, including the United Kingdom (*n* = 14), USA (*n* = 8), Canada (*n* = 6), Australia (*n* = 3) and Ireland (*n* = 2). Almost half of the studies (*n* = 14) were conducted in mental health services. Autism services made up the next biggest group (*n* = 8), with the remaining studies conducted in occupational therapy services (*n* = 2), services integrating physiotherapists and orthopaedic specialists (*n* = 3) and services broadly described as rehabilitation, early intervention or child development services (*n* = 5). All but two of the included studies described observational studies comparing waiting time for patients seen before and after an intervention, or reporting the number of people on waiting lists at two or more time points before, during or after a service redesign intervention. The exceptions were one trial involving concurrent comparison of two clinic models (Clemente et al., 2006), and one cross‐sectional survey investigating correlations between patient flow management strategies and waiting time (Vallerand & McLennan, 2013).

### 3.1 Risk of bias

Overall, there was a high risk of bias in the selected studies. No study randomised participants to intervention and comparison groups and less than half (15 of 33) used any inferential statistics to test the hypothesis that the redesign intervention was associated with a change in waiting time. Only five studies provided any information about the characteristics of the groups being compared and therefore about confounding variables, (Ahlers et al., 2019; Camden et al., 2013; Creen et al., 2021; Hine et al., 2020; Horton & Hall, 2008) and there was uncertainty about how waiting was defined or measured (i.e. the validity and reliability of outcome measures) in 14 studies (42%). A total of 29 studies did not fulfil the criterion of collecting data over the same period of time, as they used historical comparison groups, and of the four studies using concurrent comparison groups,(Clemente et al., 2006; Creen et al., 2021; Harrison et al., 2017; Horton & Hall, 2008), two were from a separate service that could not be considered the same population (Clemente et al., 2006; Horton & Hall, 2008). Further details are presented in Appendix S3. These limitations in reporting as well as heterogeneity in setting and design of the included studies meant that it was not feasible to synthesise the data using meta‐analyses.

### 3.2 The effect of service redesign on waiting for care

#### 3.2.1 Rapid response interventions

Rapid response approaches focussed on a change to the model of care in which a key component was rapid access to some form of initial appointment, usually for the purpose of engagement, prioritisation and formation of a management plan. This type of intervention was described in nine studies,(Clark et al., 2018; Clemente et al., 2006; Evans, 2014; Fuggle et al., 2016; Hayes & Caygill, 2004; Jones et al., 2018; Lynch & Hedderman, 2006; Naughton et al., 2015; York et al., 2004) all conducted in mental health services (Table 1). Three described their intervention as the Choice and Partnership Approach (CAPA), involving initial assessment at a ‘Choice’ appointment for the purpose of rapid assessment and service planning, followed by ‘Partnership’ appointments for those requiring further intervention (Clark et al., 2018; Fuggle et al., 2016; Naughton et al., 2015). The interventions described in the other six studies shared the feature of early booking for a planning or orientation session before allocation for ongoing service provision.

**TABLE 1:** Studies using rapid response strategies

Author	Setting and population	Quality criteria met (/6)	Study design	Data source		Intervention	Waiting outcome (analytic approach)	Other outcomes	Author conclusions
				Intervention data	Comparison data				
Clark et al. (2018)	Mental health service, (mean age 12 years) Canada	3	Pre post study	*N* = 154 children referred over 6 months in 2011	*N* = 794 children referred over 6 months in 2013	Choice and Partnership Approach	Time to first appointment (*t*‐test)	Time from 1st–2nd visit, patient satisfaction	Mean WT ↓ from 225 to 93 days. Small increase from 1st to 2nd appointment. High satisfaction
Clemente et al. (2006)	CAMHS, UK	3	2 service comp‐arison	*N* = 197 children at site 1 with new model of care	*N* = 204 children at site 2 (traditional model of care)	Rapid allocation to initial assessment appointments	Time to first appointment (*t*‐test)	Staff & family satisfaction, missed appointments	Mean WT ↓ from 140 to 64 days, variability and missed appt. Reduced, satisfaction increased
Evans (2014)	CAMHS, UK	1	Pre post study	*N* = 95 children	*N* = 176 children	Early access to brief initial assessment	Time to first appointment (descriptive)	Missed appointments	Observed WT ↓ 12 to 1 month, appointments missed ↓ 19% to <1%
Fuggle et al. (2016)	CAMHS, preschool to secondary age UK	3	Pre post study	*N* = 54 children referred over 6 months following intervention	Children referred over 6 months pre intervention	Choice and Partnership Approach	Time to first appointment (*t*‐test)	Waiting time variation, staff/patient experience, clinical outcomes	Mean WT from 82 to 71 days (“not significant”); reduced variability. High satisfaction, Improved outcomes against goals
Hayes and Caygill (2004)	CAMHS, UK	1	Pre post study	Waiting list post intervention; *n* = 32 low priority referrals	Waiting list 8 months prior to intervention	Rapid access to a Screening Clinic	Number on wait list (descriptive)	Appointment outcomes; satisfaction	Observed ↓ in waiting list (99 to 14 days). Half discharged after first visit. High satisfaction reported
Jones et al. (2018)	Mental health service, 0–16 years, UK	1	Pre‐post study	Waiting list 6 months post intervention; triage outcomes *n* = 200	Waiting list at baseline	“Triage clinic” providing access to rapid first assessment	Number on wait list (descriptive)	Case outcomes following triage appointment	Waiting list ↓ (200 to 56). 28% cases closed after first appointment
Lynch and Hedderman (2006)	CAMHS, Ireland	2	Pre post study	*N* = 19 children on waiting list post intervention	*N* = 62 children on waiting list pre intervention	Rapid ‘triage’ meeting; ADHD team; division of remaining waiting list	Time to first appointment (descriptive)	Number of new patients seen	Observed ↓ in mean WT (122 to 38 days). Number children assessed ↑
Naughton et al. (2015)	CAMHS, rural setting in Australia	2	Pre post study	*n* = 338 children referred 18 months prior to intervention	*N* = 134 children referred 18 months post intervention	Choice and Partnership Approach; waiting list blitz	Time to first appointment (descriptive)	Waiting time variability	Observed WT ↓ from 63.5 to 10.7 days; *SD* from 87 to 12 days.
York et al. (2004)	CAMHS, UK	1	Pre post study	Service data 6 months post intervention; *n* = 103 children seen in new clinic	Service observations pre intervention	Rapid access to first appointment	Time to first appointment (descriptive)	Patient experience	Observed WT ↓ from 8 months to 8 weeks

Abbreviations: appt, appointments; CAMHS, Child and Adolescent Mental Health Service; WT, waiting time.

**FIGURE 2:**
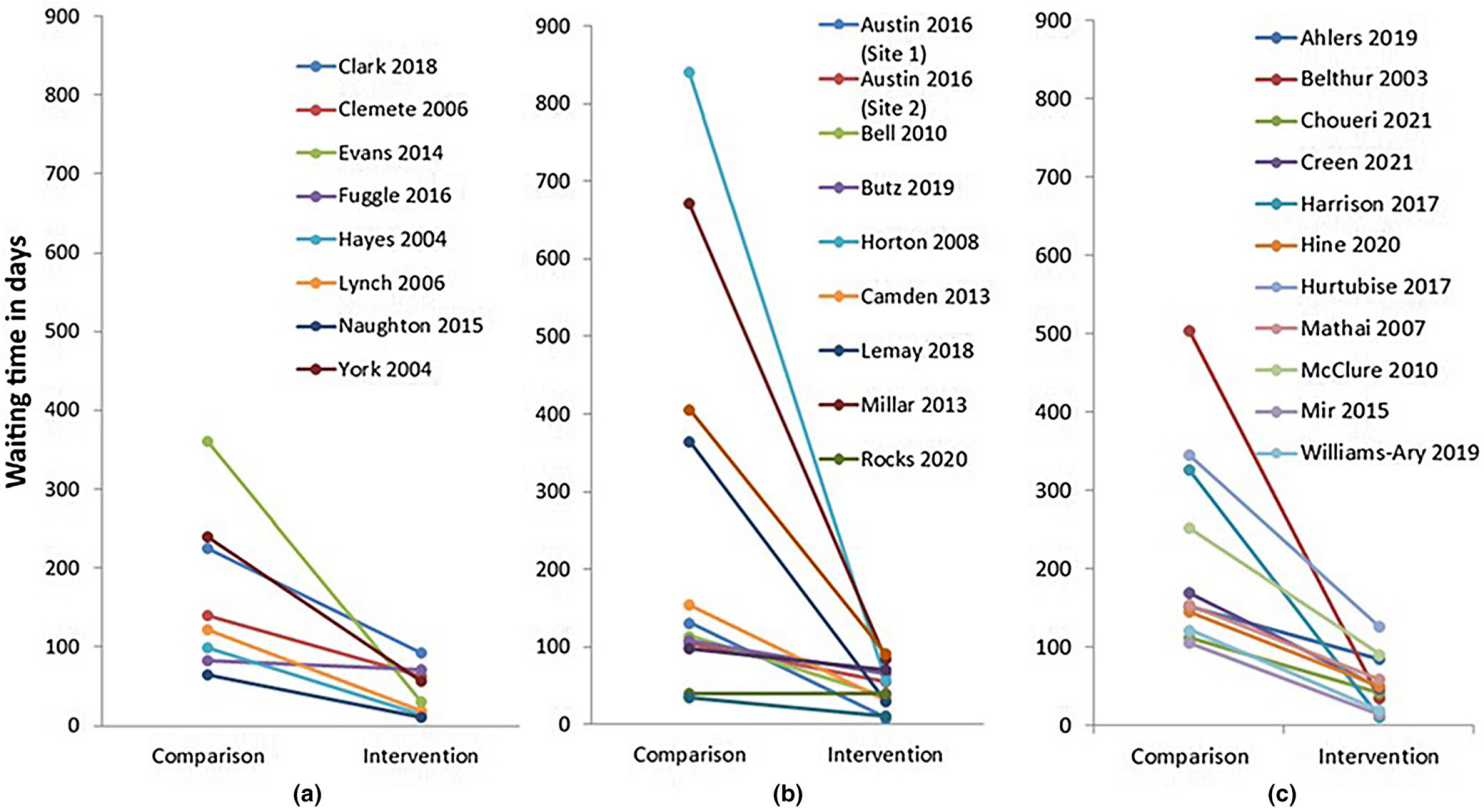
Observed changes in waiting time by intervention type. (a) Studies using early assessment strategies. (b) Studies using process redesign strategies and new care pathways. (c) Studies using substitution strategies. *Note*: Data reported in weeks or months converted to days by dividing by 7 or 30, respectively. Waiting time presented using the measure of central tendency reported by the authors (median, mean or observed typical waiting times). Only studies reporting waiting time, rather than number on a waiting list, have been included. For studies reporting multiple timepoints, data presented are a comparison between the first and last available time point.

All of the studies in this category observed reductions in either waiting time or the number of people on the waiting list (Figure 2a). However, most relied on simple observations pre and post interventions to reach a conclusion that the service redesign intervention led to improved access; only three used analytical techniques to explore the relationship between the intervention and waiting time, taking into account the variability in the data (Clark et al., 2018; Clemente et al., 2006; Fuggle et al., 2016).

#### 3.2.2 Process efficiency interventions

Process efficiency or pathway interventions used a variety of strategies to improve patient flow, such as elements of lean thinking approaches, changes to referral criteria, new patient pathways and changes to the model of care such as increased use of groups. There were 11 studies in this category (Table 2), including three in autism services (Austin et al., 2016; Lemay et al., 2018; Rutherford et al., 2018), two in occupational therapy services (Horton & Hall, 2008; F. Millar et al., 2013), four in mental health (Butz et al., 2019; Rocks et al., 2020; Wenning & King, 1995; Woodhouse, 2006) and one each in early intervention(Bell et al., 2010) and general rehabilitation (Camden et al., 2013). All but one (Rocks et al., 2020) reported reductions in waiting time (Figure 2b), with eight of these cases being reductions of more than 50%. However, with the exception of two studies (Bell et al., 2010; Camden et al., 2013), this group of studies had major methodological limitations including very little description of the characteristics or number of participants. The two studies with the lowest risk of bias showed reductions in waiting time of 69% and 80%, respectively, and both included emphasis on group treatment (Bell et al., 2010; Camden et al., 2013).

**TABLE 2:** Studies using process redesign strategies and new care pathways

Author	Population and setting	Quality criteria met (/6)	Study design	Data source intervention	Data source comparison	Intervention	Waiting outcome (analysis)	Other outcomes	Author conclusion
Austin et al. (2016)	Two autism assessment clinics (3–5 years), USA	2	Pre post study	Children referred 8 months post intervention	Children referred 3 months pre intervention	Non‐specific system redesign: identifying delays; backlog reduction; waitlist audit	Time to 3rd next appointment (descriptive)	Number on waiting list	Time to 3rd next appointment ↓ (131 to 8 days site 1, 55 to 10 days site 2). Number on waiting lists ↓ (198 to 18)
Bell et al. (2010)	Early interv‐ention (0–2.5 years), UK	3	Pre post study	Service data 2007	Service data from 2004–05	Changes to referral, assessment and case review process; ↑ groups	Time to first appointment (Wilcoxen test)	Centre caseload and group attendance	Median WT ↓ from 114 to 35.6 days. Observed increase caseload and group attendance
Butz et al. (2019)	Psychology service for pain management, USA	2	Time series	Routinely collected service data collected at monthly time points over 3.5 years		3× process improvement cycles: scheduling, waitlist management and distribution of referrals	Time to first appointment (*t*‐test)	Impact on other providers; Missed appointments	Mean WT ↓ (108 to 66 days); ↓ missed appointments (18% to 10%); no Impact on other providers
Camden et al. (2013)	Rehab servicee for children with disabilities, Canada	4	Pre post study	*n* = 188 children referred before, during and after the intervention		Service reorganisation: new admission procedures, ↑ groups, service planning with social worker	Time to first appointment (ANOVA)	Types of services offered	Mean WT↓ from 154 before to 31 days after the reorganisation. Use of groups increased
Horton and Hall (2008)	Occupational therapy service for children (0–16 years), UK	1	Pre post study	Service observations over a 3‐year period		Process redesign; backlog reduction; new care pathways; additional funding; ↑ groups	Time to first appointment (descriptive)	Complaints	WT ↓ from 2.3 years to 8 weeks. Reduction in number of complaints received
Lemay et al. (2018)	Autism assessment clinic (age 1–3 years), Canada	1	Pre post study	Service observations after 12 months	Service observations pre intervention	Redesign process: parent info sessions, screening appointment, standardised clinical pathways	Time to first appointment (descriptive)	Consumer satisfaction	WT ↓ from >1 year to <1 month. High satisfaction reported
Millar et al., (2013)	Paediatric occupational therapy service, UK	1	Time series	Annual observations of service `data over 5 years		Multi‐pronged redesign: triage system; health care support worker positions; ↑ health promotion; care agreements	Time to first appointment (descriptive)	Referral levels	WT ↓ from 96 to 12 weeks; referrals constant
Rocks et al. (2020)	CAMHS services (*n* = 3), UK	3	Pre post with control group	*N* = 57,501 spells of care over 7 years divided into pre and post intervention periods		NHS driven “Transformation initiatives”: new referral and treatment pathways and single point of access	Time to first appointment (descriptive)	Time to 2nd appointment health outcome, referral volume	Uncertain findings on WT. ↑ time to 2nd appointment, improved outcomes
Rutherford et al. (2018)	Autism assessment team, Scotland	1	Before and after	Service data 12 months post intervention	Data 12 months pre intervention	Improvement program: triage system; new treatment pathways; reduced duplication	Time to first appointment (descriptive)	Time to diagnosis; triage outcomes	WT to first assessment ↓ from 14.2 to 10.4 weeks
Wenning and King (1995)	Child psychiatric clinic, USA	1	Before and After	Service data 8 months post intervention	Service data 8 months pre intervention	Pre‐treatment orientation group as prerequisite to assessment	Time to first appointment (descriptive)		WT ↓ from 4–6 weeks to 5–10 days
Woodhouse (2006)	CAMHS, Scotland	2	Before and after	Service data reported at baseline and annually over the following 3 years		Opt in appointment system and prioritisation system that favoured treatable problems	Time to first appointment (not stated)	Rate of missed appointments	WT ↓ 58 to 13 weeks. Missed appointments reduced from 39% to 13%

Abbreviations: CAMHS, Child and Adolescent Mental Health Service; NHS, National Health Service (UK); WT, waiting time.

The nature of the intervention was also frequently poorly described or included a suite of different interventions implemented simultaneously. Common themes within the described intervention included a stronger focus on interventions conducted in groups rather than individual consultations, reduced complexity of processes, reduced variation, changes to prioritisation criteria, development of treatment pathways and workforce changes.

#### 3.2.3 Substitution interventions

Substitution interventions involved training allied health or other staff with more generic skills to provide services traditionally delivered by specialists (usually medical practitioners). Eleven studies were identified (Table 3) that evaluated interventions in which a service usually provided by a specialist was provided by other members of a healthcare team to increase treatment capacity (Ahlers et al., 2019; Belthur et al., 2003; Choueiri et al., 2021; Creen et al., 2021; Harrison et al., 2017; Hine et al., 2020; Hurtubise et al., 2017; Mathai, 2007; McClure et al., 2010; Mir et al., 2016; Williams‐Arya et al., 2019). This category included five autism services (Ahlers et al., 2019; Choueiri et al., 2021; Hine et al., 2020; McClure et al., 2010; Williams‐Arya et al., 2019) and two behavioural assessment clinics (Creen et al., 2021; Harrison et al., 2017) in which assessment roles were distributed to either trained allied health professionals or a multi‐disciplinary team, reducing dependence on specialist roles such as a psychologist or paediatrician. Three studies (two general orthopaedic services and one cerebral palsy clinic) involved physiotherapists doing work previously done by medical specialists (Belthur et al., 2003; Hurtubise et al., 2017; Mir et al., 2016). The final study trialled a change to the model of care in a child and adolescent mental health service, such that routine cases were managed by the multi‐disciplinary team with the psychiatrist taking on consultancy role rather than a personal caseload (Mathai, 2007).

**TABLE 3:** Studies using substitution strategies

Author	Setting	Quality criteria met (/6)	Study design	Intervention data	Comparison data	Intervention	Waiting outcome (analytic approach)	Other outcomes	Author conclusions
Ahlers et al. (2019)	Autism assessment service (aged 2–5 years), USA	4	Pre post study	*n* = 101 children evaluated post intervention	*n* = 143 children post intervention	Use of multi‐disciplinary clinicians rather than all children seeing psychologists	Time to diagnosis (*t*‐test)	Consumer satisfaction, diagnostic agreement	Mean time to diagnosis ↓ from 152 to 85 days. High levels of satisfaction. Strong diagnostic agreement (>93%)
Belthur et al. (2003)	Paediatric orthopaedic clinic (1–16 years), UK	1	Time series	*n* = 932 referrals, median waiting time reported annually over 4 years.		Non‐complex patients diverted from medical consultation to specialist physiotherapy clinic	Time to first appointment (descriptive)	Cost, patient perception	WT ↓ from 72 to 5 weeks. High satisfaction. Reduced costs for physio clinic
Choueiri et al. (2021)	Autism Clinic for toddlers in underserviced community, USA	0	Cohort study	*n* = 81 children screened by EI staff	‘Usual care’ source not described	Screening by trained EI workers	Time from screening to diagnosis (descriptive)	Agreement between EI providers and psychologist	Time to diagnosis ↓ (16 to 6 weeks). Moderate correlation between EI workers and psychology assessment.
Creen et al. (2021)	Regional child development service, Australia	5	Pre post study	Record audit post inter‐vention (*n* = 75); 2 years (*n* = 75).	Random sample (*n* = 75) 6 months pre intervention	Advanced practice Allied Health practitioners (OT and psychologist) added to clinic	Time to first appointment (Kruskall–Wallis)	Time to diagnosis; % referrals not needing medical referral	Median WT ↓ (169 to 48 days) and time to diagnosis (59 to 31 days). 52% no requiring medical input
Harrison et al. (2017)	Developmental Behavioural Clinic for children <5 years, USA	4	Cohort study	*n* = 63 children seen in new clinic	Time to next usual care appointment (same cohort)	Multidisciplinary Behavioural Developmental Access Clinic (BDAC)	Time to first appointment (*t*‐test)	Number of people on waiting list, need for specialist review	Mean WT ↓ (327 to 159 days) for patients accepting BDAC appointment, less need for specialist evaluation
Hine et al. (2020)	Integrated health care clinics providing autism assessment, USA	3	Pre post study	Chart review pre‐intervention (*n* = 40)	Chart review post‐intervention (*n* = 40)	Screening by trained Behavioural Health Consultants (BHCs)	Time from first concern to diagnosis (descriptive)	Number of children requiring further specialist assessment	Time to diagnosis reduced from 144.7 (*SD* 99.9) to 49.9 (*SD* 32.2) days. 9/40 required additional assessment
Hurtubise et al. (2017)	Clinic for cerebral palsy surveillance, Canada	3	Pre post study	Referrals in 3 years post change	Referrals in 12 months pre change	Children seen by advanced practice physiotherapist instead of orthopaedic surgeon	Time to first appointment (descriptive)	Time to review appointments	WT ↓ from 345 to 127 days; review time reduced from 764 to 15 days
Mathai (2007)	CAMHS, Australia	1	Pre post study	Service observations after 12 months	Service observations at baseline	Change in model—psychiatrist having consulting role only, MDT carry active caseloads	Time to first appointment (descriptive)		WT ↓ 22 to 8.4 weeks
McClure et al. (2010)	Autism assessment service, UK	1	Pre post study	*n* = 38 children assessed post intervention	*n* = 38 children referred prior to change	Substitution of specialist assessment with local teams (various disciplines)	Time to first appointment (*t*‐test)	Agreement on diagnostic decisions	Mean WT ↓ (38 to 13 weeks). High‐level agreement between local teams and specialists
Mir et al., (2016)	Paediatric orthopaedic clinic, Ireland	3	Pre post study	Clinic data “pre 2010”	*n* = 2650 patients seen over first 3 years of clinic	Physio specialist clinic—diversion from medical consultant clinics	Time to first appointment (descriptive)	Clinical outcomes following assessment	WT ↓ (102 to 15 weeks). 21% subsequently referred to orthopaedic clinic after physio assessment
Williams‐Arya et al. (2019)	Autism clinic, USA	2	Time series	Monthly clinic data over 3 years		Multi‐disciplinary team assessment rather than behavioural paediatrician alone	Time to first appointment (descriptive)	Total visits required, treatment cost	Median WT ↓ (122 to 19 days), total visits reduced (5 to 2), per patient cost reduced

Abbreviations: CAMHS, Child and Adolescent Mental Health Service; EI, early intervention; NHS, National Health Service (UK); OT, Occupational Therapy; WT, waiting time.

All but one (Ahlers et al., 2019) of the studies that evaluated substitution services reported waiting times or number of people on the waiting list were reduced by 50% or more (Figure 2c).

#### 3.2.4 Other strategies

Two studies (Vallerand & McLennan, 2013; Wittmeier et al., 2016) that met the criteria for this review did not test an intervention in one of the above categories (Table 4). One study trialled a central intake service for multiple services for children with complex needs, reporting a small observed reduction in waiting time from 12 to 8 days for complex referrals, and minimal change for children with orthopaedic conditions (Wittmeier et al., 2016). A second study conducted a cross‐sectional survey of 113 child and adolescent mental health agencies (Vallerand & McLennan, 2013) and looked for associations between clusters of demand management strategies and waiting time. Use of ‘upstream’ strategies (intake processes, triage, prevention and early intervention) showed mild correlation with reduced wait time for low‐ and medium‐priority patients, but there were no significant effects for other types of strategies.

**TABLE 4:** Studies using other interventions

Author	Setting	Quality criteria met (/6)	Study design	Intervention data	Comparison data	Intervention	Waiting outcome (analytic technique)	Other outcomes	Author conclusions
Vallerand and McLennan (2013)	CAMHS, Canada	3	Cross sectional survey	Questionnaire to 379 agencies, 113 responses		Clusters of different strategies to improve access to care	Time to first appointment (Spearman's rank coefficients)	Ability to meet targets	No relationship found between clusters of strategies and WT
Wittmeier et al. (2016)	Rehabilitation service, Canada	3	Before and After	*n* = 1399 children with complex needs and 3901 with orthopaedic concerns		Central intake system	Time to first appointment (Kruskall–Wallis test)		Median WT ↓ (12 to 8 days) for children with complex needs, and small rise (9.6 to 10.1 days) for orthopaedic referrals

Abbreviations: CAMHS, Child and Adolescent Mental Health Service; WT, waiting time.

### 3.3 Secondary outcomes

Nine of the 33 studies reported on consumer satisfaction (Ahlers et al., 2019; Belthur et al., 2003; Clark et al., 2018; Clemente et al., 2006; Fuggle et al., 2016; Hayes & Caygill, 2004; Horton & Hall, 2008; Lemay et al., 2018; York et al., 2004). Of these, most authors simply reported high levels of satisfaction following the intervention without comparison to the comparison condition. However, one study reported an increase in satisfaction after introducing a rapid response model (Clemente et al., 2006) compared to usual care and another study measured a decrease in complaints following a process redesign intervention (Horton & Hall, 2008).

Rates of missed appointments were reported in two studies using rapid response interventions (Clemente et al., 2006; Evans, 2014) and two studies using process redesign interventions (Butz et al., 2019; Woodhouse, 2006). Both reported reductions in the proportion of people who missed appointments. Three studies reported the time to second appointment (Clark et al., 2018; Hurtubise et al., 2017; Rocks et al., 2020); there was a dramatic reduction in time to review appointments with a substitution model, (Hurtubise et al., 2017), but studies using a process redesign approach (Rocks et al., 2020) and a rapid response approach (Clark et al., 2018) observed small increases in time to the second appointment. In both of the latter cases, the overall time from referral to second appointment was still reduced following the intervention, suggesting that any delays from the first to second appointment were outweighed by faster access to a first appointment. Cost effectiveness was considered in two studies, both substitution services, and identified cost savings associated with the new model of care (Belthur et al., 2003; Williams‐Arya et al., 2019).

Two studies measured clinical outcomes, both conducted in a child and adolescent mental health services. Introduction of a rapid response model found no reduction in effectiveness of treatment following the intervention, and some improvement measured against the intervention goals (Fuggle et al., 2016). Introduction of ‘transformation initiatives’ driven by the UK National Health Services in three regions found some evidence to suggest that outcomes may have been improved following the interventions, but highlighted that confounding variables may have influenced these findings (Rocks et al., 2020).

Some additional observational data were reported that provided some additional insights into the impact of the service redesign strategies. Qualitative data regarding staff satisfaction were reported in two studies in the rapid response category; both reported the changes to have had benefits in relation to collaboration, but noted concerns about increased time pressures from some participants (Clemente et al., 2006; Fuggle et al., 2016). Six studies considered services offered following an initial appointment; two studies measured the proportion of children needing further evaluation after initial assessment using a rapid response model (Hayes & Caygill, 2004; Jones et al., 2018) and four studies reported the proportion requiring further assessment by a specialist physician (Creen et al., 2021; Hine et al., 2020; Mir et al., 2016; Rutherford et al., 2018) after initially seeing an alternative/substitution professional. All reported that clinically significant numbers of children could be discharged without requiring further care. Three studies evaluated agreement between specialists and alternative providers making diagnostic decisions in substitution interventions, and all reported moderate‐to‐high agreement between clinicians (Ahlers et al., 2019; Choueiri et al., 2021; McClure et al., 2010). Finally, two process redesign studies reported the intervention to be associated with increased use of groups (Bell et al., 2010; Camden et al., 2013), two reported increases in patients seen post intervention (Bell et al., 2010; Lynch & Hedderman, 2006) and one checked for any adverse impacts of scheduling changes for other service providers not involved in the intervention but found no effect (Butz et al., 2019).

### 3.4 Certainty of the evidence

**FIGURE 3:**
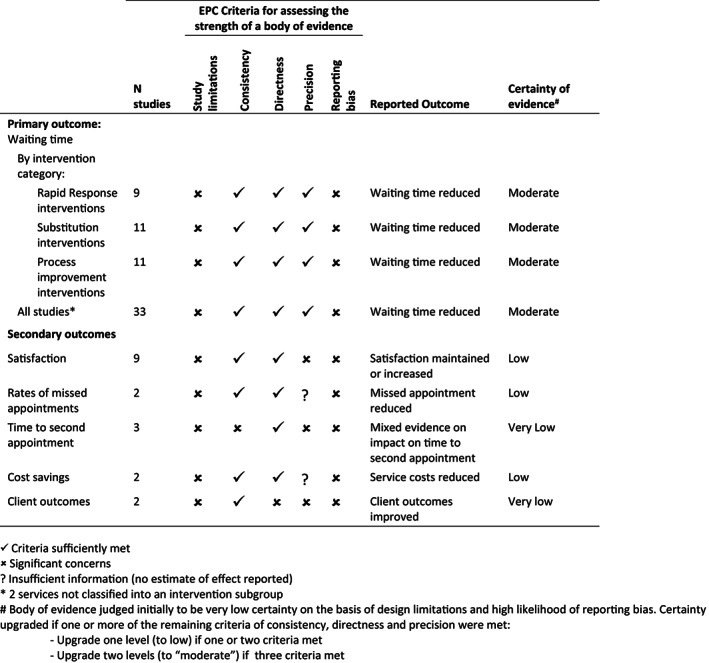
Certainty of evidence summarised according to Evidence‐based Practice Centre (EPC) criteria (Berkman et al., 2015).

Using the EPC guidelines (Berkman et al., 2015), overall, this body of evidence offers moderate‐certainty evidence that service redesign interventions can reduce waiting time in paediatric rehabilitation and therapy services. The evidence is based on multiple studies with a high degree of study limitations and high likelihood of reporting bias (Figure 3). However, these limitations are mitigated by the consistency of findings across studies, direct evidence in relation to the primary outcome and large estimates of effect. This evaluation is consistent across the three types of interventions and the body of literature. The body of evidence for secondary outcomes offers low to very low‐certainty evidence due to the relatively small number of studies considering each outcome, inconsistency of findings in relation to some outcomes and limited reporting.

## 4 DISCUSSION

This review of 33 studies investigated the effectiveness of service redesign interventions to reduce waiting time in paediatric rehabilitation and therapy settings. The interventions were classified into three major categories: models of care promoting rapid access to a first appointment; general redesign initiatives that sought to increase efficiency or modify care pathways; and enabling/substituting allied health or other professionals to deliver services previously provided by medical specialists. The vast majority of included studies reported more timely access to care following the intervention, with many reporting reductions in either time to first appointment or the number of people on a waiting list by more than 50%.

The consistent evidence with large estimates of effect in reducing waiting time from more than 30 studies provides moderate‐certainty evidence that reductions in waiting time can be achieved with service redesign interventions in paediatric settings. Long waiting lists for these services have been reported in multiple countries (Harding et al., 2017; A. R. Millar, 2008; Porterfield & McBride, 2007), with serious consequences for child development and well‐being of families. When waiting is an entrenched and pervasive problem, it can easily begin to be seen as inevitable, or at least a problem that can only be fixed through a large injection of additional resources (Harding et al., 2017). The evidence in this review challenges this notion, suggesting that service redesign interventions, which by their nature do not typically require costly resources, can make a difference.

Despite these positive findings, conclusions are limited by the high risk of bias in the majority of included studies. Most of the studies were observational studies, with comparisons made with historical controls. Many were designed as quality improvement initiatives, intended to achieve local results rather than demonstrate internal validity. This was reflected in the reporting: 85% failed to report on the characteristics of the sample, and only 45% conducted any statistical analysis to provide an estimate of confidence around the reported result. Of those that did report inferential statistics, parametric statistics were commonly used to test differences in waiting time without reference to the distribution of the data. This is potentially problematic given that waiting time data are often positively skewed (Siciliani et al., 2014). It is also highly likely that this field of study is subject to reporting bias; services that have been successful in improving access to care have been keen to share their achievements, but initiatives that have not produced the desired results may have been left unreported.

The three categories of interventions identified in this review were favoured by different types of services. Strategies that provided access to a rapid initial assessment appointment were used mainly by mental health services. Substitution services were trialled mainly in autism and orthopaedic services. The group of studies testing interventions that involved reducing inefficiencies and/or modifying aspects of the patient pathway were featured in a more diverse range of settings. None of the three categories of interventions showed clear benefit over the others, and heterogeneity within each category suggests that were also likely contextual factors such as the size of the original waiting list, the type of service provided, characteristics of the local population or organisational factors that influenced the outcome. However, findings suggest that all three categories of intervention are feasible in paediatric services.

While the impact of redesign strategies on waiting times was the primary focus of this review, many of the included studies also considered other outcomes. Once again, the evidence is limited by the nature of the studies, but no adverse outcomes were reported over a very broad range of secondary outcomes. Under a variety of circumstances, benefits were reported in reducing failure‐to‐attend rates and decreased costs. Several studies checked for adverse impacts on factors such as time to second appointment, accuracy of assessment, patient satisfaction, clinical outcomes and impact on staff not involved in the intervention, but none reported any cause for concern as a result of the interventions. These findings suggest that interventions that reduce waiting time do not necessarily require trade‐offs with other aspects of care delivery, and may have additional benefits. However, the studies in this review primarily focussed on time to the first appointment, and two of the three studies that did measure time to review appointments noted a modest increase following the intervention (Clark et al., 2018; Rocks et al., 2020). Concerns about ongoing caseload management have also been raised by staff involved in implementation of similar initiatives in adult services (Harding et al., 2019). Further research is needed to understand the long‐term impacts of redesign initiatives on ongoing access to care, particularly given the chronic nature of many of the conditions seen in paediatric rehabilitation and therapy settings.

Paediatric rehabilitation and therapy services were the subject of this review because this population has unique features that we hypothesised could influence the choice or effectiveness of interventions to reduce waiting time. For example, services for children may need to accommodate timing around specific developmental stages, integration with the education system or the involvement of families in the care of the child. However, findings of this review suggest that the strategies trialled to reduce waiting time in paediatric settings are similar to those published in services for adult populations. Success in reducing health service waiting times has been reported for adult populations from substitution services (Desmeules et al., 2012), interventions providing rapid access to first appointments (Harding et al., 2018; Murray & Berwick, 2003) and process redesign approaches (van Leijen‐Zeelenberg et al., 2016). The only significant point of contrast in the paediatric literature to literature from adult settings was the absence of any ‘walk‐in’ clinic models (Handley et al., 2018); this could reflect a higher level of complexity in paediatric care or perceived need for forward planning for families or may represent an area for future research in paediatric rehabilitation. Overall, this review suggests that paediatric and adult services do not need to be considered fundamentally different when considering methods to reduce waiting lists and provide more timely access to care. Evidence‐based interventions developed in one setting are likely to be adaptable to others.

This review has some limitations. Developing a successful search strategy for the concept of waiting time was challenging, due to the broad nature of terms such as ‘wait’ and ‘time’. The matrix approach used in this review, based on proximity searches, is imperfect and may have missed some relevant papers. However, citation and reference list checking resulted in only two additional included articles, suggesting the strategy achieved its purpose and was sufficiently sensitive. Determining what should be included under the definition of ‘therapy’ used in this review was not always straightforward. In particular, autism services varied considerably in the extent to which they provided a medical diagnostic service only versus ongoing support and therapy. Given that the nature of these services was not always well documented, we used the presence of allied health professionals in a multi‐disciplinary team as the indicator for inclusion.

The lack of robust studies with a low risk of bias is a clear limitation of this body of literature, and may be explained by a perception that these types of interventions, usually implemented at a service level, are not appropriate for testing with randomised controlled trial designs. While it is true that individual randomisation may not be possible or practical due to factors such as the risk of contamination, other trial designs such as cluster trials or interrupted time series approaches are viable alternatives, and have been successfully applied to these types of interventions in other settings (Handley et al., 2018; Portela et al., 2015). Where pre/post designs are the only viable option, risk of bias could still be substantially reduced by the inclusion of a control site, reporting characteristics of the pre and post intervention cohorts, and subjecting findings to appropriate statistical analyses (Portela et al., 2015). Improving the quality of research in this field would provide service providers with more robust evidence on which to base decisions about investing resources in initiatives to improve access.

## 5 CONCLUSION

There is moderate‐certainty evidence that service redesign interventions, including substitution services, rapid access to a first appointment and process redesign initiatives, can improve timely access to care in paediatric rehabilitation and therapy services.

## AUTHOR CONTRIBUTIONS

All authors contributed to the conceptualisation and design of the study. Harding conducted the search of bibliographic databases for the review. All authors contributed to screening and selection of studies, and interpretation and validation of findings. Harding prepared the original draft of the manuscript, and all authors contributed to editing and review.

## CONFLICT OF INTEREST

The authors have no interests to declare.

## Supporting information


Appendix S1.

## Data Availability

Data sharing is not applicable to this article as no new data were created or analysed in this study.
