# Supplementary material for: Service redesign interventions to reduce waiting time for paediatric rehabilitation and therapy services: A systematic review of the literature
Source: Health Soc Care Community. 2022 Jun 18;30(6):2057–70. doi: 10.1111/hsc.13866 (PMC10084082; doi:10.1111/hsc.13866)
Supplement: Supplementary file 1 — Appendix S1. [file HSC-30-2057-s001.docx]

**Supplementary File**

**Appendix A: Search Strategy in Medline**

| 1 | child*.mp. OR Child/ OR pediatric.mp. OR Pediatrics/ OR paediatric.mp. OR youth.mp. |
| --- | --- |
| 2 | early intervention.mp. OR "Early Intervention (Education)"/ OR Rehabilitation/ OR rehabilitation.mp. OR special need.mp. OR handicap*.mp. |
| 3 | Allied Health Personnel/ OR Allied Health Occupations/ OR Allied Health.mp. OR physiotherap*.mp. OR Speech-Language Pathology/ OR speech$language pathol*.mp. OR Dietetics/ OR dietetic*.mp. OR Dietary Services/ OR dietitian*.mp. OR prosthet*.mp. OR ORthot*.mp. OR Psychology/ OR psycholog*.mp. |
| 4 | autism.mp. OR Autistic Disorder/ OR Cerebral Palsy/ OR Developmental Disabilities/ OR Intellectual Disability/ OR disability.mp. OR Disabled Persons/ OR Attention Deficit Disorder with Hyperactivity/ OR Mental Health/ OR Learning Disorders/ OR developmental dyspraxia.mp. OR Motor Skills Disorders/ OR motor coordination disORder.mp. OR (sensory adj3 disorder).mp. OR brain injury.mp. OR Brain Injuries/ OR head injury.mp. OR Craniocerebral Trauma/ OR Arthritis, Juvenile/ OR Dyslexia, Acquired/ OR Dyslexia/ OR Down Syndrome/ OR spina bifida.mp. OR Spinal Dysraphism/ OR Dyscalculia/ OR Vision Disorders/ OR vision impair*.mp. OR Hearing Loss/ OR Deafness/ OR hearing impair*.mp. |
| 5 | "wait*".m_titl. OR (appointments and schedules).mp. |
| 6 | (wait* adj time).mp. OR (Wait* adj5 length) OR (Wait* adj5 duration) OR (Wait* adj5 size) OR (Wait* adj5 number) OR (Access adj5 delay).mp. OR (Wait* adj5 access) OR time to care.mp. |
| 7 | 2 OR 3 OR 4 |
| 8 | 5 OR 6 |
| 9 | 1 AND 7 AND 8 |

**Appendix B: Table of inclusion and exclusion criteria**

Inclusion/exclusion criteria

|  | **Inclusion Criteria** | **Exclusion Criteria** |
| --- | --- | --- |
| **Population/ setting** | Services provided in outpatient/community settings  AND  Providing rehabilitation/therapy services (involvement of allied health professionals accepted as an indicator of rehabilitation/therapy)  AND  Focussing on paediatric populations, including adolescents if part of a mixed paediatric population. | Services provided in inpatient settings (e.g. hospitals, residential rehabilitation)  Services focussed only on older adolescents (e.g. 16+) and transition to adulthood  Services limited to outpatient medical consultations only. |
| **Interventions** | Any redesign intervention implemented at the service level to improve patient flow, including but not limited to changes in:   - Management of waiting list - Referral or triage processes - Personnel providing services - Scheduling processes - Care models (e.g. individual vs group treatment or new care pathways) - The allocation of resources | Studies that did not involve a redesign intervention. For example:  - studies looking at factors associated with long waiting times  - studies considering the impact of waiting times on patient outcomes |
| **Outcomes** | Studies reporting on an outcome related to waiting, expressed either as:   - Time to first appointment - Time to important milestone in patient journey (e.g. time to diagnosis or specialist appointment) - Size of waiting list | Studies that do not include data for an outcome related to waiting time.  Time in waiting room |
| **Study design** | Any design reporting comparative data on timeliness of care under different service conditions, including but not limited to:   - Historically controlled trials - Randomised controlled trials - Observational studies comparing outcomes in different settings - Cluster designs - Quality improvement projects - Mixed methods studies provided quantitative data reported on primary outcome | - Studies that describe service redesign initiatives without comparative data on the primary outcome - Opinion/discussion papers - Reviews |
| **Sources** | Peer review journal articles | Conference abstracts  Book chapters  Reports |
| **Language** | Papers published in English or French | Papers published in other languages |

**Appendix C: Summary of risk of bias across all studies and decision rules for included items from the Downs and Black Quality Checklist**

**Risk of Bias assessment items were selected from:**

Downs SH, Black N. The feasibility of creating a checklist for the assessment of the methodological quality both of randomised and non-randomised studies of health care interventions. Journal of epidemiology and community health. 1998 52(6):377-84.

Six of eleven internal validity items included in the scale were selected for this study. The items chosen were those that were most relevant to the study designs in the selected studies:

- whether participants were randomised to intervention and comparison groups
- whether groups being compared were recruited from the same population
- whether groups being compared were recruited over the same time period
- whether appropriate statistical tests were used to evaluate findings
- whether outcomes measures were valid and reliable
- whether confounding variables had been considered in the analysis.

Other internal validity items on this checklist were considered to be less useful in differentiating the quality of studies in this review due to the nature of the interventions being studied. For example, waiting list interventions are usually applied to a whole service and those receiving the service are often unaware that a trial is in progress, thus limiting the applicability of concepts such as blinding of participants and compliance with the intervention

**Decision rules for the 6 included items:**

**Item 1: Were participants randomised to intervention and comparison groups?** Awarded “yes” if randomisation had taken place, either at individual or cluster level.

**Item 2: Were groups being compared were recruited over the same time period?** Awarded “no” for historically controlled trials, including before and after and time series designs.

**Item 3: Were groups being compared recruited from the same population?** Awarded “yes” if participants had been drawn from the same population. For example, recruitment from within the same service, or comparison across two or more services with demonstration of equivalence of the population demonstrated

**Item 4: Were appropriate statistical tests were used to evaluate findings?** Awarded “no” where no statistical tests had been conducted or tests selected were inappropriate

**Item 5: Were confounding variables considered in the analysis? A**warded “yes” if characteristics of the intervention and comparison groups had been documented and either

- - No differences between groups that would be likely confounders were identified or
  - Any differences identified had been considered in the analysis

Awarded “no” if characteristics of the intervention and comparison groups were not described, or differences were not considered in the analysis.

**Item 6: Were outcomes measures valid and reliable?** Awarded “yes” if the measurement of waiting was clearly defined. For example, a clear definition of who was included in the count of people on a waiting list, or the definition of ‘waiting’ such as the time from the date of referral to first face to face appointment with a clinician.

Supp Figure 1: Selected validity items from the Down’s and Black Checklist assessed as having been met in the included studies


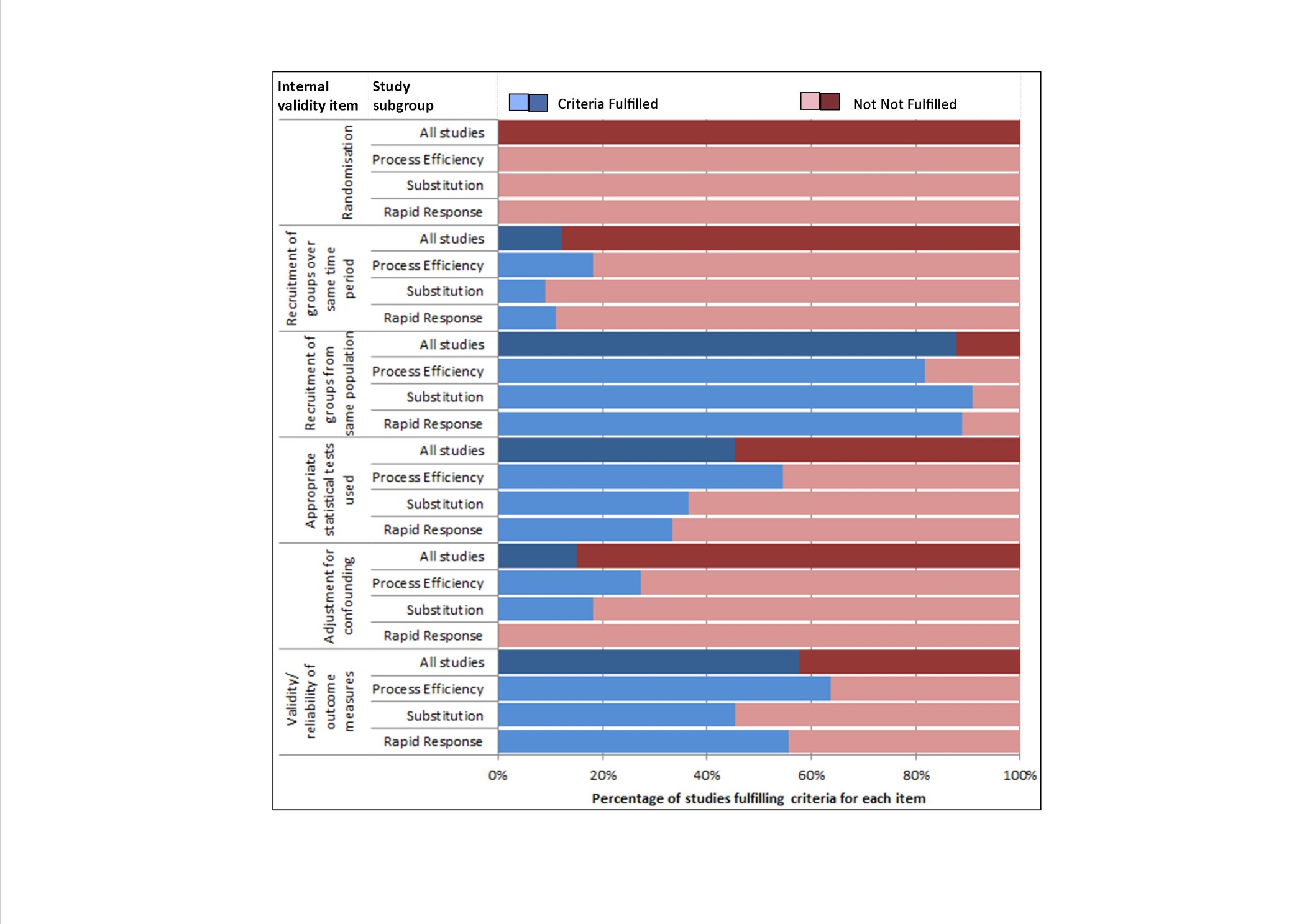


| *Percentage of all studies (n=33) filling criteria* |  |
| --- | --- |
|  |  |
| *Percentage studies within each intervention subgroup filling criteria* |  |
| *(Process efficiency n=11, substitution n=9, rapid response n=11,   not classified n=2)* |  |
